# The Cholera in Englaud

**Published:** 1848-11

**Authors:** 


### THE MEDICAL EXAMINER.

PHILADELPHIA, NOVEMBER, 1848.

### THE CHOLERA IN ENGLAND.

We have had repeated rumours within the past few weeks of the appearance of this scourge in England, but at last, we regret to say, we have it announced in a way that leaves no room for doubt, that it has manifested itself in various places.

The steamer Europa, which arrived at New York on the 24th ult., having sailed from Liverpool on the 10th, brings the authentic intelligence. The following extract from Wilmer & Smith's Journal, will show its progress up to the date of the steamer's departure:

"The Cholera in England.—We regret to state that the scourge which, during the last few months, has desolated the eastern parts of

Europe, spreading its ravages from Cairo to St. Petersburg, and lingering within these few weeks at Hamburgh, has at length, as anticipated, reached the shores of Great Britain. It is now officially declared by the Registrar-General that the Asiatic Cholera has appeared in the metropolis, and well authenticated cases of the malady are reported from Sunderland, Shields, Hull, and Edinburgh. The disease made its appearance almost contemporaneously in Sunderland and in the low lying districts below London Bridge. In both places the first cases were those of intemperate sailors who came from Hamburgh, and were attacked by the malady on the voyage. As regards Edinburgh, the origin of the disease is left in doubt.

"The official report of the Registrar-General in London, reported 13 cases up to Saturday last. In Edinburgh, up to the latest report, there had been 25 cases, 20 of which had proved fatal. Up to Wednesday in the present week, the number of cases in London is alleged to be about 20, but a daily official report is not yet issued. The authorities in all parts of the country seem to be taking the most zealous precautions to counteract, prevent, and remedy this dreadful malady, which we earnestly hope will make but a brief visit to our shores. The alarm is greatly diminished respecting its destructive effects amongst the great body of the people, and we trust, with the extensive arrangements made to check its progress, that the limits of its mortality will be confined to the seaport towns, and that the great manufacturing hives of industry will be spared this frightful addition to the many sufferings they have lately experienced."

